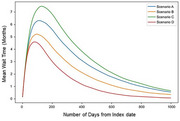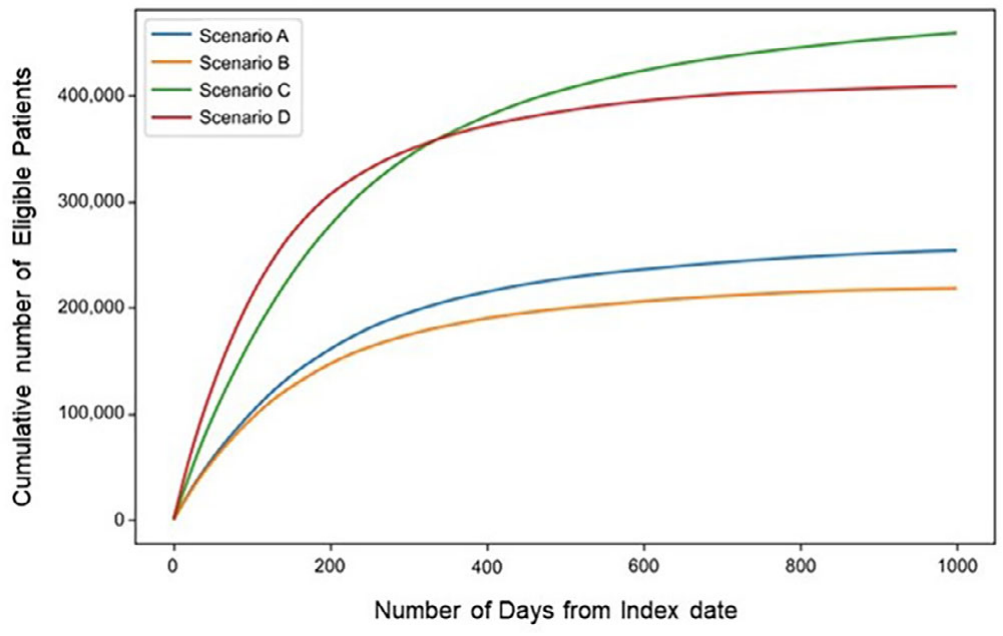# Impact of Blood‐Based Biomarkers on Access to Alzheimer's Disease Treatments: A Simulation Study in Japan

**DOI:** 10.1002/alz70860_102553

**Published:** 2025-12-23

**Authors:** Ataru Igarashi, Noriyuki Kimura, Temmei Ito, Chizuru Kobayashi, Kotaro Sasaki, Yukinori Sakata, Mie Azuma, Etsuro Matsubara

**Affiliations:** ^1^ The University of Tokyo, Graduate School of Pharmaceutical Sciences, Tokyo, Japan; ^2^ Oita University, Yufu, Oita, Japan; ^3^ Eisai Co. Ltd., Tokyo, Japan; ^4^ Eisai Co., Ltd., Tokyo, Japan; ^5^ Faculty of Medicine, Oita University, Oita, Japan

## Abstract

**Background:**

New disease‐modifying therapies (DMTs) targeting amyloid‐beta (Aβ) for mild cognitive impairment and mild dementia due to Alzheimer's disease (AD) currently require brain Aβ confirmation using amyloid positron emission tomography (PET) or cerebrospinal fluid (CSF) biomarkers. Although diagnosis of AD using widely available blood‐based biomarkers (BBM) would be desirable, their impact on the medical care system has not been fully investigated. In this study, we estimated wait times for diagnostic testing and the number of patients eligible for the DMTs by converting wait times, cost, and test characteristics across various scenario for AD diagnosis flow incorporating BBM into monetary value.

**Method:**

A dynamic simulation model was used for estimations. For the simulation, the demand for testing was estimated from an online survey of 3,302 normal participants based on their willingness to pay (WTP) for the testing according to assigned cognitive impairment severity. The simulation was conducted under four scenarios: (A) the current diagnostic flow, (B) implementing the BBM test as a triage test, (C) implementing the BBM test as a confirmatory test, and (D) implementing the BBM test both for a triage in primary care physician (PCP) setting and as a confirmatory test in specialist setting.

**Result:**

WTP varied with cognitive impairment severity; mild dementia had the highest mean WTP at JPY 22,810 (standard deviation: JPY 57,209). The maximum patients average wait times for diagnostic ranged from 4.7 months to 7.8 months across the scenarios, with peak wait times being longest in the order of Scenarios C, A, B, and D (Figure 1). The estimated cumulative number of patients eligible for the DMTs were larger in Scenarios C and D compared to Scenarios A and B: 431,357, 394,830, 230,231, and 211,356, respectively, by 2025 (Figure 2).

**Conclusion:**

The findings suggest that implementing BBM for a triage test demonstrated potential for reducing wait times, particularly in PCP settings. Additionally, utilizing BBM as a confirmatory test can increase the number of treatment‐eligible patients, primarily due to increased specialist capacity and decreased diagnostic cost associated with BBM, suggesting that this approach could notably improve access to timely treatment.